# Childhood obesity and central precocious puberty

**DOI:** 10.3389/fendo.2022.1056871

**Published:** 2022-11-18

**Authors:** Li Shi, Zhiyan Jiang, Li Zhang

**Affiliations:** ^1^ Department of Pediatrics, Longhua Hospital, Shanghai University of Traditional Chinese Medicine, Shanghai, China; ^2^ Institute of Digestive Diseases, Longhua Hospital, Shanghai University of Traditional Chinese Medicine, Shanghai, China

**Keywords:** childhood obesity, central precocious puberty, metabolic status, integration, prevention strategies

## Abstract

Childhood obesity is a major public health problem worldwide, and the relationship between obesity and central precocious puberty has long been confirmed, however, the mechanisms underlying this association remain elusive. This review provides an overview of the recent progress regarding how childhood obesity impacts on hypothalamic-pituitary-gonadal axis and pubertal onset, focusing on adipokines (leptin and ghrelin), hormone (insulin), and lipid (ceramide), as well as critical signaling pathways (AMPK/SIRT, mTOR) that integrate the peripheral metabolism and central circuits. Notably, prevention of obesity and CPP is beneficial for the adult life of the children, thus we further summarize the potential strategies in treating and preventing childhood obesity and CPP. The updated understanding of metabolic stress and pediatric endocrine disease will arise the attention of society, and also contribute to preventing more serious comorbidities in the later period of life in children.

## Introduction

Precocious puberty refers to the early onset of puberty, manifests as early secondary sexual characteristics and physical development, and is a pediatric endocrine disease. Precocious puberty can be mainly classified as central precocious puberty (CPP) and peripheral precocious puberty (PPP) according to whether the hypothalamic-pituitary-gonadal (HPG) axis is activated. The initiation of the HPG axis is usually considered true precocious puberty, therefore, CPP is the dominant diagnosis. The development of secondary sexual characteristics before the age of 8 in girls and 9 in boys is defined as CPP. CPP shows an obvious gender dimorphism, which is a conserved feature of puberty in higher mammals, possibly due to environmental and psychological factors. A recent systematic review and meta-analysis indicated that pooled prevalence is 25% in girls less than 6 years of age, although significant heterogeneity exists in different age groups (1). Females have a prominent population of Kiss1 neurons in the anterior ventral periventricular nucleus, which is crucial for establishing the positive feedback between ovarian steroids and the gonadotropin-releasing hormone (GnRH) surge generator, facilitating the females more sensitive to the central regulatory effects of some metabolic signals than in males (2, 3).

Detailed descriptions of the HPG axis in controlling puberty can be found in many excellent reviews. In brief, GnRH neurons in the hypothalamus release GnRH pulse to the hypophyseal portal blood system, while the gonadotroph cells in the pituitary respond to the signal and release the gonadotrophins luteinizing hormone (LH) and follicle-stimulating hormone (FSH), which reach the gonads to drive puberty onwards. Therefore, the evaluation of LH peaks after GnRH testing is the gold standard in the biochemical diagnosis of CPP. The combined detection of secondary sexual characters, bone age, hormone levels, pelvic ultrasound and cranial MRI could increase the sensitivity and accuracy for CPP diagnosis (4, 5).

The development of CPP is both determined by genetic and environmental factors. The past decade has confirmed a series of core genes of CPP, such as the kisspeptin gene (KISS1), the kisspeptin receptor gene (KISS1R), the makorin ring finger protein 3 (MKRN3), and the Delta-like non-canonical Notch ligand 1 (DLK1). It is reported that DLK1 gene mutation is associated with the prevalence of family CPP, and MKRN3 gene loss-of-function mutations are the most prevalent genetic etiology of CPP. Apart from rare genetic variants, the occurrence of metabolic comorbidities rises as the dominant cause of CPP in recent years. The epidemic of childhood obesity serves as the major companion for CPP, suggesting the impact of nutritional and metabolic cues on the HPG axis. There is clear evidence to support the effects of higher childhood body mass index (BMI) on the onset and development of puberty in both boys and girls (6). Childhood obesity and PP are both important risk factors for metabolic syndrome, type 2 diabetes, and insulin resistance (7). Genome-wide association study (GWAS) in humans identified multiple BMI-increasing alleles that are also associated with earlier age of menarche, further confirming genetic co-regulation of childhood obesity and CPP (8). Notably, early timing of puberty directly affects the adult height, muscle, and fat mass accrual, induces various psychological symptoms, conduct problems, and is also linked to an increased risk of cardiovascular events, breast cancer, higher susceptibility to attention deficit-hyperactivity disorder ADHD (9), as well as various oncologic, cognitive, and behavioral disorders, and even reduced life expectancy (10–12). Therefore, PP has raised global public health concerns, and strategies that prevent PP are highlighted worldwide.

## Metabolic basics of CPP

Reproduction is an energy-intensive, but essential process for the survival of all species. Therefore, a certain threshold of body fat storage is required to initiate and maintain reproductive function. Overweight women have served as fecundity icons dating from *Palaeolithic* Ages. Whereas individuals with anorexia or malnutrition are often related to delay or absence of puberty onset and perturbed fertility (13). The current generation of adolescents is growing up at a time of unprecedented ample food, whereby nutritional deficiency and food insecurity are eliminated, and overweight and obesity are burgeoning. Nutrition overwhelming in modern society not only causes metabolic burden, but also accelerates the process of puberty. Studies showed that BMI could affect the GnRH stimulation test, and the peak of LH stimulation is negatively correlated with BMI (14, 15).

The metabolic-reproductive interplay emphasizes the link between body energy reserves and reproductive function, and lays the basis for the impact of different metabolic disorders, ranging from anorexia to obesity and metabolic syndrome, on puberty and fertility. Hypothalamic circuits are responsible for the tight coupling between body energy status and puberty onset. Among these circuits, GnRH neurons operate as the final output pathway for the central control of the onset of puberty, and a plethora of metabolic hormones and neuropeptides are coupled and tightly regulated the function of GnRH neurons. Elimination of the inhibition on the HPG axis is dynamically balanced before the initiation of puberty. Interaction of kisspeptin and kisspeptin receptor, synchronized operation of Neurokinin-B, glutamate, leptin, and androgens are considered to be drivers of GnRH pulse generation, whereas endogenous opioid peptides such as dynorphin A, gamma-aminobutyric acid (GABA), MKRN3 are acted as inhibitors of GnRH release (16).

Clinical and experimental work further provide evidence to support the interaction of energy reserves and CPP. The age of menarche was approximately 17 years in the early 19th century, while it dramatically declined to approximately 13 years by the mid-20th century, largely attributed to improved nutrition, personal hygiene, and better socioeconomic conditions (6). Studies in different regions all reported that increased BMI is a predictor of early onset of puberty in girls (17–20). Notably, body composition is found to be more sensitive to earlier puberty onset than BMI (21). Furthermore, higher birthweight is also a predictor of younger ages at menarche and advanced breast development, and gestational glucose intolerance is associated with increased odds of offspring overweight/obesity in late adolescence (22, 23). Notably, children born small for gestational age are also prone to develop obesity, metabolic syndrome, as well as CPP, which might be explained by thrifty phenotype hypothesis (24, 25). Collectively, these findings further confirmed the link of metabolic status and CPP.

## Effects of adipokines and hormones on CPP

Obesity is accompanied by a series of metabolic alterations, and different metabolic cytokines and hormones, such as leptin, ghrelin, insulin, as well as certain central lipids may impact the HPG axis, and participate in the fine-tuning of puberty.

### Leptin and ghrelin

Leptin is the first identified adipose cytokine, which is a peptide hormone (16kDa) encoded by the product of the obese (*ob*) gene, and secreted from the adipocytes into the circulation, the level of leptin is directly related to the amount of body fat stores. Leptin can pass the blood-brain barrier, and take the action at certain neurons in the hypothalamus. Physiologically, circulating leptin is a sensitive maker of metabolic status, and transports the signal to the control center (hypothalamus). Upon binding with leptin receptor (LepRb), leptin activates pathways such as JAK2/STAT3, PI3K/IRS/AKT, and SHP2/MAPK in the hypothalamus, and exerts anorexigenic and thermogenic functions to alleviate the metabolic burden in the peripheral. Simultaneously, the accumulation of phosphorylated STAT3 dimers induces the transcription of SOCS3, which inhibits the JAK2/STAT3 pathway. This efficient work of the leptin signaling feedback loop guarantees metabolic homeostasis. Accordingly, leptin deficiency (*ob/ob* mice) and leptin receptor deficiency (*db/db* mice) animals spontaneously develop into obesity and/or type 2 diabetes.

In addition to metabolic control, leptin is also a permissive factor for the activation of GnRH neurosecretion at puberty. Kisspeptins that are produced by hypothalamic Kiss1 neurons are fundamental GnRH regulators, and leptin deficiency has been found to decrease hypothalamic Kiss1 expression, whereas exogenous administration of leptin increases Kiss1 in rodent models of leptin deficiency (26). The clinical investigation reported that the serum leptin level is obviously higher in CPP girls than in the controls (27). Consistently, individuals with malnutrition have low levels of leptin with a delay in puberty onset. Leptin acts as the upstream afferent signal for GnRH neurosecretion, whereas GnRH neurons that lack functional LepRb require alternative afferent pathways to perform their regulatory actions.

Obese individuals exhibit high leptin concentrations due to the adipose tissue expansion, however, the high leptin concentrations failed to obtain the expected suppression of food intake and increased energy expenditure, a phenomenon that is termed leptin resistance (28). Leptin resistance is a hallmark of obesity, featuring a high concentration of circulating and central leptin. The lack of feedback of leptin signal in leptin resistance might persistently stimulate kiss1 expression, and destroy the balance of NKB and Dyn modulation on kisspeptin secretion (29).

Contrary to the anorectic action of leptin, the gut-derived peptide ghrelin is required for the orexigenic process. In both mice and humans, increased appetite is correlated with elevated levels of circulating ghrelin. Ghrelin directly acts upon the HPG axis by stimulating adreno-cortico-tropic-hormone (ACTH) synthesis and secretion in the anterior pituitary, or indirectly elevates ACTH through paracrine stimulation of hypothalamic corticotropin-releasing hormone synthesis and secretion. It is reported that plasma ghrelin is negatively correlated with BMI and body fat percentage, and circulating ghrelin levels are decreased in human obesity (30). Consistently, a progressive reduction in ghrelin levels has been observed during puberty, and GnRH analog (GnRHa) treatment in CPP girls further decreases the circulating ghrelin levels (31). However, the reason why ghrelin secretion decreases at puberty is not yet known.

Every other day fasting (EODF) is reported to delay the Di-(2-ethylhexyl) phthalate-induced puberty onset acceleration in female rats, accompanied by the decrease of serum leptin, luteinizing hormone and estradiol (32). Considering the locations of the central action of leptin and the Kiss1 neurons are consecutive and possibly overlapped, the accumulation of metabolic hormone in the hypothalamus might be an important integrator to explain childhood obesity and CPP.

### Insulin

Insulin is another important hormone that affects both childhood obesity and CPP. Insulin is a pancreatic hormone, and secreted in response to the increase of blood concentration of glucose (hyperglycemia), to promote circulating glucose to enter effector cells. Reduced sensitivity of cells to insulin is termed insulin resistance, which often occurs in obesity and related T2DM. The inefficient glucose uptake in conditions of insulin resistance causes hyperglycemia, which further stimulates insulin secretion to compensate the insulin insensitivity. However, continuous hyperglycemia and insulin resistance in obesity has been shown to conduct stimulatory/permissive actions on the HPG axis. Actually, conditions of low or null insulin levels, such as uncontrolled diabetes, are usually associated with suppressed GnRH levels and reproductive activity (33). Mice lacking insulin receptors selectively in neurons are obese and show a delay in development due to GnRH deficiency (34). On the contrary, high insulin levels in female rodents and women show significantly increased LH secretion (35, 36).

During development, nutrient consumption promotes growth as well as the production of required hormones through insulin-like systems (37). The hormonal profile in peri-pubertal girls with obesity is characterized by hyperinsulinemia and higher HOMA-IR index, especially evident in early puberty (38). Upon obesity and insulin resistance, the compensated insulin secretion might accelerate the timing of puberty. In a cross-sectional study that included 79 girls with CPP and 37 girls with premature thelarche, Li et al. found that the serum insulin-like growth factor-1 (IGF-1), IGF binding protein-3 (IGFBP-3) levels are obviously a higher in CPP girls compared with controls (39). Metformin is a widely used drug for treating T2DM and is also used for delaying sexual maturation in girls with CPP. Heterogeneous mice (UM-HET3) that were treated with metformin (i.p) between the ages of 15 and 56 days showed increased insulin sensitivity and normal sexual maturation in female pups, indicating insulin signaling and puberty are tightly integrated (40). Insulin also stimulates the synthesis of leptin in adipocytes, the two hormones might synergistically modulate puberty onset.

## Molecular link of metabolic and reproductive circuits on CPP

The identification of *Kiss1*, and its receptor Gpr54 is considered to be a breakthrough in puberty. *Kiss1* governs the secretion of kisspeptin, which directly acts on GnRH neurons *in promoting* puberty activation. The Kiss1 system is sensitive to metabolic conditions, and an important transmitter for impacting puberty. Therefore, hypothalamic kisspeptin neurons have been postulated to be a key nodal nexus between metabolism and puberty (41). Childhood obesity is usually associated with an active Kiss1 system, and a series of molecular substrates are involved in this process.

### AMPK/SIRT signaling

AMPK is a fundamental nutrition sensor that is essential for cellular energy homeostasis, alteration of cellular AMP/ATP ratio determines the status of AMPK. Upon energy deficiency, an increased AMP/ATP ratio directly activates AMPK *via* phosphorylation at Thr-172 of the α-subunit, attempting to restore the balance. Exercise is an efficient way to deplete energy, and the AMPK is kept in active status during exercise. In contrast, an overwhelming energy supply (including childhood obesity) will inhibit AMPK activity.

AMPK is widely expressed in metabolic organs and also co-expressed in Kiss 1 neurons, therefore the brain AMPK is likely to integrate metabolic/nutritional status and the onset of puberty in obesity. Chronic energy deficiency at puberty activates hypothalamic AMPK, and pharmacological or virogenetic activation of AMPK delays pubertal onset to a variable extent in rodent models (42). AMPK is found to inhibit the Kiss 1 gene, thus suppressing the function of GnRH neurons (43). On the contrary, childhood obesity inhibits AMPK activity (44), which might relieve the suppression of the Kiss 1 gene, and contribute to the development of CPP. Hypothalamic activation of AMPK along with the persistence of the repressive action of SIRT1 at the Kiss1 promoter, leads to reduced Kiss1 expression, whereas the eviction of SIRT1 from the Kiss1 promoter as well as AMPK suppression in conditions of overnutrition, transactivates Kiss 1 gene transcription and the GnRH secretion (45, 46).

Central AMPK activity also regulates peripheral metabolism. Estrogens (E2) are reported to inhibit hypothalamic AMPK through estrogen receptor alpha (ERα), which leads to the promotion of thermogenesis in brown adipose tissue in a feeding-independent manner. Genetic activation of AMPK in the VMH prevented E2-induced increase in brown adipose tissue-mediated thermogenesis and weight loss (47).

### mTOR signaling

Mammalian target of rapamycin (mTOR) is atypical serine/threonine protein kinase, and is an evolutionally conserved protein. mTOR can integrate multiple intracellular signals (nutrition, energy, and growth factors), and is involved in the transcriptional and translational regulating processes.

In conditions of energy sufficiency, timely eviction of SIRT1 from the Kiss1 promoter, together with the presumable activation of mammalian target of rapamycin (mTOR), allows increased Kiss1 expression and the normal occurrence of puberty. Activation of PI3K/Akt/mTOR pathway in the hypothalamus is associated with the increased GnRH release and CPP in adolescent female rats, whereas inhibition of mTOR in the hypothalamus could block the activation of Kiss1, Grp54, and GnRH (48). In addition, activation of mTOR also induces Akt phosphorylation and kisspeptin release, which also contributes to GnRH secretion and CPP (49). The central concentration of kisspeptin also affects the mTOR pathway, it is reported that Kisspeptin 10 (Kp-10) maintains the activation of mTOR signaling (50). Accordingly, pharmacological inhibition of kisspeptin weakens mTOR pathway (51). Therefore, the interaction of mTOR and kisspeptin might further drive the initiation of CPP.

### Hypothalamic ceremide

A central ceramide signaling pathway serves as an alternative mediator of childhood obesity and CPP. Different from kisspeptin’s direct regulation of GnRH neurons, hypothalamic ceramide involves the paraventricular nucleus (PVN) and sympathetic ovarian innervation. Obese female rats with CPP show higher expression of serine palmitoyltransferase long-chain base subunit 1 (SPTLC1), a crucial component for *de novo* ceramide synthesis, the increased ceramide level further results ovarian sympathetic output, whereas blockade of ceramide synthesis normalized the timing of puberty and ovarian sympathetic tone (52). Consistently, another report revealed that early-onset obesity enhanced ceramide synthesis in PVN, which accelerates the maturation of the ovarian noradrenergic system, virogenetic suppression of SPTLC1 that inhibits ceremide synthesis, in turn reverses obesity-induced CPP, indicating that central ceremide is crucial in integrating metabolic and neuronal circuits in CPP (53).

## Treatment and prevention strategies for CPP

Puberty is a crucial biological process normally occurring at a specific time, therefore, the aim of CPP treatment is to normalize the course of puberty, preserve the adult height, and alleviate the associated complications. GnRH secretion is considered the initial drive for the development of CPP, therefore, GnRH analog (GnRHa) is the primary option for CPP. GnRHa treatment shows efficacy in suppressing gonadotrophin and slowing the progression of secondary sexual characteristics (54). However, whether the GnRHa treatment benefit adult height is still in conflict, and treatment with GnRHa has different effects on BMI according to baseline body composition (55). Patients with CPP are frequently obese due to hormonal and metabolic changes, and a decrease in BMI has been reported in these patients during GnRHa therapy (56). A study tracked 92 adult females that implemented GnRHa treatment in childhood, and found that these subjects had normal BMI and body composition, although the final height is not increased consistently (57). Another study evaluated the effects of GnRHa treatment in 94 girls with idiopathic CPP, and reported increased insulin resistance but normal BMI and lipid profile over 2 years after menarche (58). On the contrary, a study has followed up body composition longitudinally in girls show a gradual increase in adiposity, a decrease in muscle mass, and bone mineral density during GnRHa treatment, whereas bone mass was preserved after treatment (59). In patients with hypothalamic hamartoma, GnRHa treatment increases the mean BMI and the percentage of body fat mass in females, a possible reason is that GnRHa treatment causes an increase in appetite and consequently an elevation of the fat mass index in CPP girls (60, 61). Collectively, GnRHa therapy has a favorable effect on CPP, whereas further well-designed longitudinal investigations are needed to evaluate its long-term metabolic outcomes.

Prevention is the first and foremost strategy for CPP and obesity. A healthy lifestyle is crucial for the development of children. Away from “junk food” should be highlighted. Overconsumption of high-fat high-fructose food is the driving force of obesity, especially in children. It is reported that high-fructose corn syrup consumption (e.g., drinks and desserts) during childhood has stronger metabolic effects than in other generations, which is closely associated with CPP (62). An animal study revealed that EODF can delay puberty onset acceleration in female rats (32), indicating appropriate diet restrictions can be taken into consideration. In addition to diet control, exercise is another important approach for the prevention of CPP in children (63). Lack of out-door exercise may affect insulin sensitivity and vitamin D levels. A systematic meta-analysis comparing serum vitamin D levels between patients with CPP and controls revealed that vitamin D-deficient subjects are more likely to develop CPP, suggesting that CPP may be linked to vitamin D deficiency (64). However, a cross-sectional study fails to observe significant differences in serum 25(OH)D concentration between CPP girls and prepubertal controls (65). Although there are inconsistent findings in different reports, a recent trial reports that the frequency of CPP cases increased approximately three times during the COVID-19 pandemic due to prolonged stress, home quarantine, as well less excise (66). CPP and obesity also affect calcium-phosphate metabolism and adult height, therefore, adequate outdoor exercises are strongly recommended, and adequate sunshine and vitamin D are beneficial for children in all aspects.

Guaranteeing the quality and duration of sleep of the children is equally important for preventing obesity and CPP. The circadian timing system regulates a variety of biological actions including metabolism, hormone, immunity, and reproductive function. Sleep quality and duration determine the hypothalamic melatonin secretion, which impacts the transcription of kisspeptin and GnRH (67). In addition, sleep also promotes the secretion of growth hormones and attribute to the prevention of ADHD. Moreover, attention should also be paid to the psychological status of the children, avoiding negative emotions is one of the effective strategies in managing a healthy lifestyle for children.

Exogenous chemicals that interfere with the endocrine system are defined as endocrine disruptors. Endocrine disruptors may affect the synthesis, metabolism, and the action of endogenous hormones, leading to the dysregulation of normal physiological processes and promoting the development of disease (57). Endocrine disruptors are ubiquitous in the environment, and might also be risks for CPP. In a case-control study, Zhou et al. compared urinal metabolites and serum hormones of 30 precocious puberty girls with 46 age- and race-matched prepubertal females, and confirmed the association of CPP with phthalate esters exposure (68). Ubiquitously present bisphenol A is another threat to female CPP, possibly through Kiss1 activation in ARC (69, 70). Children that are exposed to antibiotics, especially fluoroquinolones and tetracyclines are reported to be positively associated with the occurrence of CPP (71). Pesticides that extensively used in farming act as endocrine-disrupting chemicals, and significantly affect the time of puberty onset (72). In addition, pheromones are also considered to promote hormone secretions and induce CPP (73). Collectively, keeping children from this environmental pollution helps prevent CPP and related diseases.

## Conclusions and perspective

**Figure 1:**
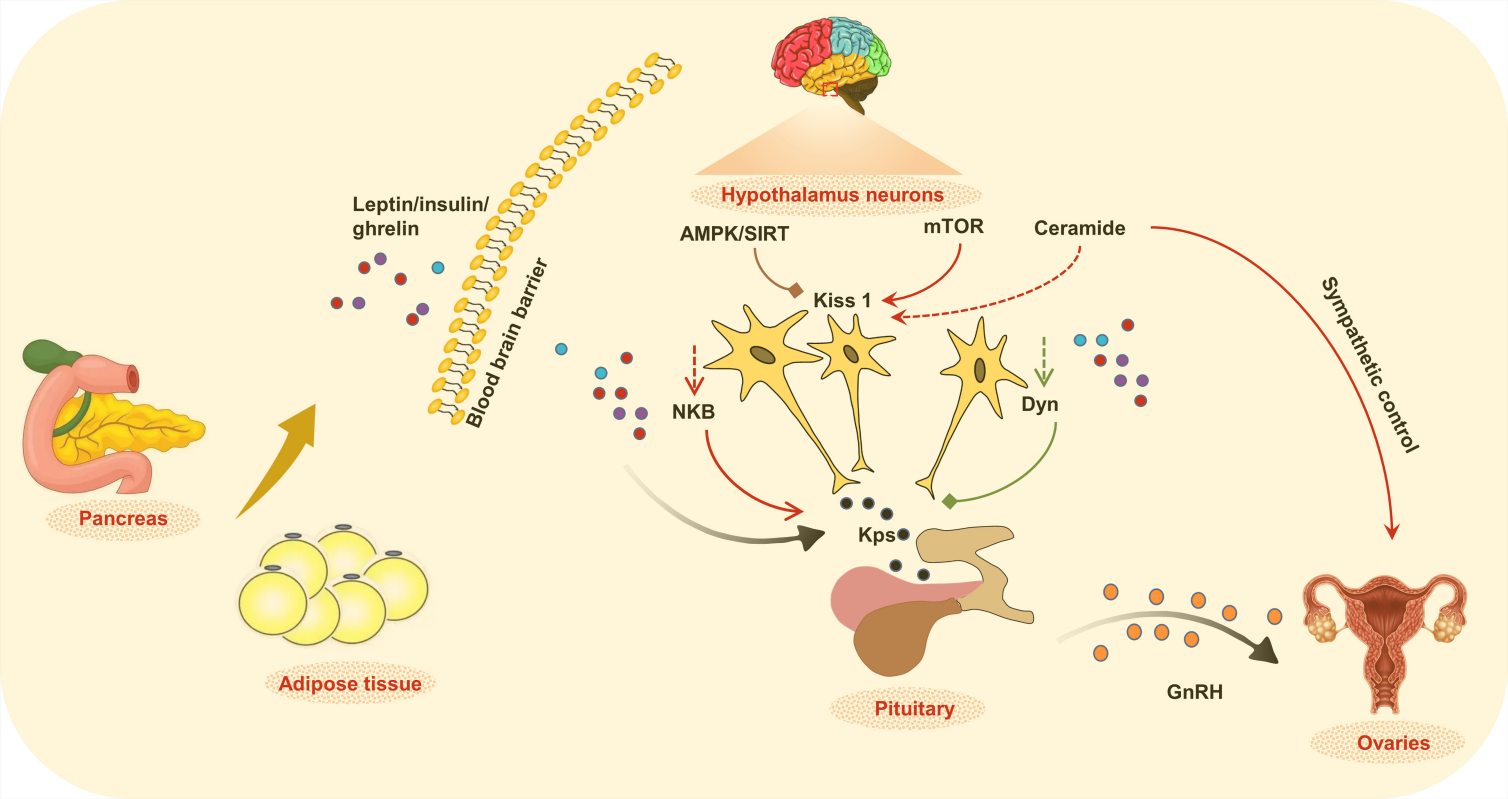
The regulation of metabolic and endocrine functions. Alteration of peripheral metabolic status in childhood obesity changed the production and release of cytokines, hormones, lipid, as well as energy sensitive molecules that integrate the control of metabolic signals and central circuits, which together affect the initial of puberty.

**Figure 2:**
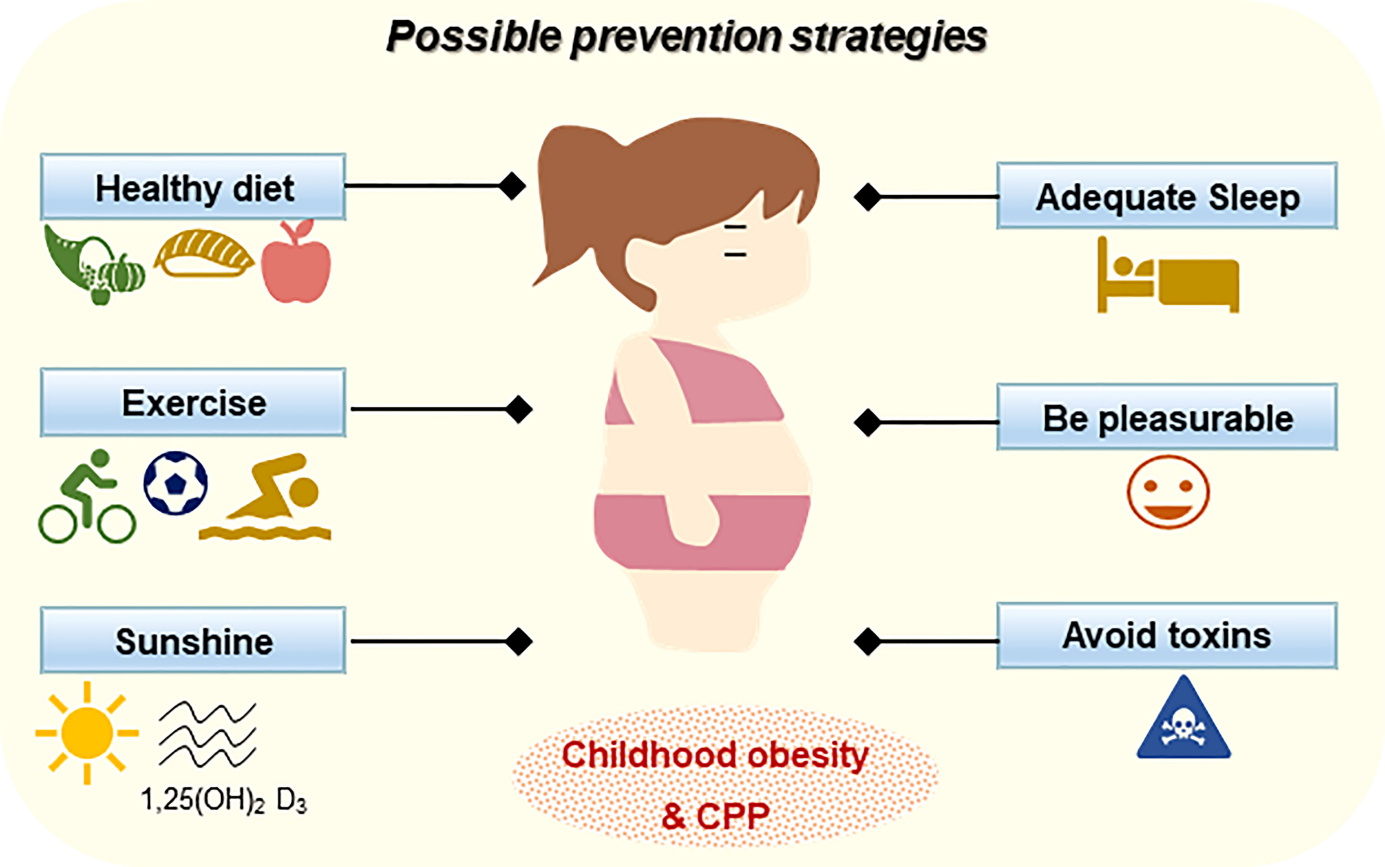
The possible prevention strategies for childhood obesity and CPP.

The obesity epidemic and CPP are associated with a series of metabolic and endocrine diseases, and might also impact the quality of life in adults. In this review, we have focused on the peripheral cytokines, hormones, lipids, as well as energy-sensitive molecules to integrating the metabolic and endocrine functions during CPP (**Figure 1**). Understanding the role of childhood obesity in CPP will draw attention to treating and preventing the disorder in children. Considering the complex interaction of the two systems, and the genetic, psychological and environmental impact on CPP, we propose that prevention should be considered to be the foremost strategy for childhood obesity and CPP (**Figure 2**). Maintaining physical and mental health, as well as a safe environment is not only beneficial for children not only at adolescence, but also for whole life health.

## Author contributions

LZ conceptualized the manuscript, LS and ZJ collected the literature and drafted the manuscript, LZ revised the manuscript. All authors contributed to the article and approved the submitted version.

## Funding

This work was supported by grants from the National Natural Science Foundation of China (No. 82205181).

## Conflict of interest

The authors declare that the research was conducted in the absence of any commercial or financial relationships that could be construed as a potential conflict of interest.

## Publisher’s note

All claims expressed in this article are solely those of the authors and do not necessarily represent those of their affiliated organizations, or those of the publisher, the editors and the reviewers. Any product that may be evaluated in this article, or claim that may be made by its manufacturer, is not guaranteed or endorsed by the publisher.
